# Quercetin supplementation alters adipose tissue and hepatic transcriptomes and ameliorates adiposity, dyslipidemia, and glucose intolerance in adult male rats

**DOI:** 10.3389/fnut.2022.952065

**Published:** 2022-09-29

**Authors:** Adéla Kábelová, Hana Malínská, Irena Marková, Martina Hűttl, Blanka Chylíková, Ondřej Šeda

**Affiliations:** ^1^First Faculty of Medicine, Institute of Biology and Medical Genetics, General University Hospital, Charles University, Prague, Czechia; ^2^Center for Experimental Medicine, Institute for Clinical and Experimental Medicine, Prague, Czechia

**Keywords:** metabolic syndrome, quercetin, retroperitoneal fat, glucose intolerance, insulin, triglycerides

## Abstract

Quercetin, a flavonoid present in many fruits and vegetables, exhibits beneficial effects toward abnormalities related to metabolic syndrome. In this study, to further investigate metabolic and transcriptomic responses to quercetin supplementation, we used a genetic model of metabolic syndrome. Adult male rats of the PD/Cub strain were fed either a high-sucrose diet (HSD; control PD rats) or HSD fortified with quercetin (10 g quercetin/kg diet; PD-Q rats). Morphometric and metabolic parameters, along with transcriptomic profiles of the liver and retroperitoneal fat, were assessed. The relative weights of epididymal and retroperitoneal fat were significantly decreased in quercetin-treated animals. Furthermore, a smaller area under the glycemic curve along with a decreased level of fasting insulin were detected in PD-Q rats. While no changes in total cholesterol levels were observed, the overall level of triglycerides decreased in the serum and the liver of the PD-Q rats. The transcriptomic profile of the liver and the adipose tissue corroborated the metabolic and morphometric findings, revealing the pattern consistent with insulin-sensitizing changes, with major regulator nodes being *Pparg*, *Adipoq*, *Nos2*, and *Mir378*. In conclusion, quercetin supplementation improves abnormalities related to metabolic syndrome, namely adiposity, dyslipidemia and glucose intolerance.

## Introduction

Metabolic syndrome (MetS) represents a cluster of metabolic abnormalities including central obesity, glucose intolerance, hypertension, and atherogenic dyslipidemia, which together greatly increase the risk for cardiovascular disease or type 2 diabetes (1, 2). Although there have been various attempts to identify mechanisms for the underlying pathophysiology of MetS, the complex and multifaceted origin of the syndrome is yet to be fully understood (3). The most widely accepted hypothesis suggests insulin resistance/hyperinsulinemia as the main pathogenic mechanism (3, 4). However, other mechanisms, such as central (visceral) adiposity, oxidative stress, and low-grade chronic inflammation, have also been proposed to be involved in the development and/or progression of the syndrome (5–7).

In recent years, MetS has become one of the main public health concerns associated with enormous social, personal, and economic hardship (8). As the incidence of the syndrome increases worldwide, effective intervention strategies for treating MetS are needed (9). The management of MetS is usually based on lifestyle changes, particularly in one’s dietary habits, but must be frequently accompanied by pharmacological treatment (10). However, current medication rather aims at treating individual components of MetS, (e.g., using hypolipidemic, antihypertensive or antidiabetic drugs), not addressing the complex nature of the disease (11). A multi-functional drug that would ameliorate multiple features of the syndrome does not yet exist to our knowledge.

One of the most widely distributed nutraceuticals in the human diet is a flavonoid quercetin as it can be found in various fruits, vegetables, nuts, seeds, and tea (12). Numerous *in vitro* and *in vivo* studies have demonstrated quercetin’s favorable impact on obesity, dyslipidemia, glucose intolerance, and hypertension, as well as oxidative stress and inflammation that both seem to be involved in the development and progression of the MetS (13–15). This ability to target numerous components of MetS makes quercetin a highly potent agent offering a potentially effective treatment alternative or an add-on to standard pharmacotherapy (16–18).

In this study, we examined the effect of oral quercetin administration on morphometric and metabolic parameters associated with MetS as well as the transcriptomic profiles of the liver and retroperitoneal fat tissue. For this purpose, we used an inbred rodent model of MetS that carries a variant in the *Zbtb16* (Zinc finger and BTB Domain Containing 16) gene that is known to modulate the propensity for features of MetS including adipogenesis, insulin sensitivity and dyslipidemia in both humans and rodent models (19, 20).

## Materials and methods

### Ethical statement

All experiments were performed in agreement with the Animal Protection Law of the Czechia and were approved by the Ethics Committees of the First Faculty of Medicine of the Charles University (Permit Number: MSMT-19427/2019-8).

### Rat strains

The polydactylous rat [PD/Cub, Rat Genome Database ID 728161] is a highly inbred strain originated from a polydactylous pair of random-bred Wistar rat strain. The strain has not only been exploited as a genetic model of limb malformation (21, 22) but was established as a model for MetS as it carries a variant in one of the metabolic syndrome-related genes, *Zbtb16* (23, 24). In addition, the PD/Cub strain was repeatedly shown to be particularly sensitive to sucrose diet-induced dyslipidemia and insulin resistance (23, 25). Since 1969, the PD/Cub rat strain has been maintained at the Institute of Biology and Medical Genetics by brother x sister mating.

### Experimental protocol

Adult male rats of the PD/Cub (PD hereafter) strain were held under temperature- (23°C) and humidity- (55%) controlled conditions on 12-h light/12-h dark cycle and fed a laboratory chow diet (STD, ssniff RZ, ssniff Spezialdiäten GmbH, Soest, Germany). At all times, the animals were given free access to food and water. At the age of 12 months, the animals were randomly divided into two groups (*n* = 6/group). Over the period of 2 weeks, the control group was fed a high-sucrose diet (HSD, protein (19.6 cal%), fat (10.4 cal%), carbohydrates (sucrose, 70 cal%) prepared by Institute for Clinical and Experimental Medicine, Prague, Czechia; PD rats), (26) while the experimental group was fed a HSD fortified with quercetin (10 g quercetin/kg food) (Sigma-Aldrich; PD-Q rats). The dose of quercetin used here was chosen to be similar to the doses used in previous studies (16, 27). Bodyweight and food intake were measured twice a week for each group.

Blood samples for metabolic and glycemic assessments were drawn after overnight fasting from the tail vein. For the oral glucose tolerance test, blood samples were obtained at intervals of 0, 30, 60, 90, 120, and 180 min after intragastric glucose administration to conscious rats (3 g/kg body weight, 30% aqueous solution). Blood glucose concentrations over the period of 180 min were used to calculate the area under the curve. All rats were then sacrificed and the weight of heart, liver, kidneys, adrenals, and brown, epididymal and retroperitoneal adipose tissue were determined. Biochemical parameters were determined as follows: serum total cholesterol and triglycerides concentrations using kits from Erba Lachema (Brno, Czechia); non-esterified fatty acids using kit from Roche Diagnostic (Mannheim, Germany); insulin using a rat insulin enzyme-linked immunosorbent assay kit (Mercodia, Uppsala, Sweden) and high-molecular weight (HMW) adiponectin using an ELISA kit (MyBioSource, San Diego, CA, USA). To determine triglycerides and cholesterol in the liver tissue, samples were extracted in chloroform/methanol. The resulting pellet was dissolved in isopropyl alcohol with the content of the triglycerides determined by enzymatic assay (Erba-Lachema, Brno, Czechia).

### Transcriptomic analysis

Total RNA was isolated from the liver and retroperitoneal fat (RNeasy Mini Kit, Qiagen, Hilden, Germany). The quality and integrity of the total RNA were evaluated on Agilent 2100 Bioanalyzer system (Agilent, Palo Alto, CA, USA), and only samples with RNA Integrity Number (RIN) >8.0 were utilized in further steps of the protocol. Microarray experiments were performed using the Rat Gene 2.1 ST Array Strip in triplicate for each group/tissue combination, i.e., total of 12 arrays were processed. The hybridization procedure was performed using the Affymetrix GeneAtlas system according to manufacturer’s instructions. The quality control of the chips was performed using Affymetrix Expression Console software (Affymetrix, Santa Clara, CA, USA). Partek Genomics Suite 7 (Partek, St. Louis, MO, USA) was used for subsequent data analysis. After applying quality filters and data normalization by Robust Multichip Average (RMA) algorithm, the set of obtained differentially expressed probe sets was filtered by the false discovery rate (FDR) method that is implemented in Partek Genomics Suite 7 (Partek, St. Louis, MO, USA). Only probe sets with FDR <0.1 and, at the same time, showing >1.2fold or <−1.2fold difference in expression between the control and experimental group were subjected to further analyses.

Transcriptomic data were then processed by a sequence of analyses (hierarchical clustering and principal component analysis, gene ontology, gene set enrichment, upstream regulator analysis, mechanistic networks, causal network analysis and downstream effects analysis) using Ingenuity Pathway Analysis (Qiagen). The microarray data generated and analyzed during the current study are available in the ArrayExpress repository^1^ under accession number E-MTAB-11061.

### Quantitative real-time PCR

To validate the gene expression data obtained by microarray, quantitative real-time PCR (RT-qPCR) was performed. The amount of 1 μg of total RNA was used to synthesize cDNA using oligo-dT primers and the SuperScript III reverse transcriptase (Invitrogen, Carlsbad, CA, USA). For validation, the following sets of TaqMan probes (Thermofisher; Waltham, MA, USA) were used: ras homolog family member T1 (*Rhot1*): Rn01751396_m1, tsukushi, small leucine rich proteoglycan (*Tsku*): Rn01506888_g1, acetyl-CoA carboxylase alpha (*Acaca*): Rn00573474_m1, ATP citrate lyase (*Acly*): Rn00566411_m1, Stonin 1 (*Ston1*): Rn00788269_m1, regulated endocrine specific protein 18 (*Resp18*): Rn00570625_m1, fatty acid desaturase 1 (*Fads1*): Rn00584915_m1, 7-dehydrocholesterol reductase (*Dhcr7*): Rn00574366_m1. RT-qPCR reaction was performed in triplicate with TaqMan Gene Expression Master Mix (Applied Biosystems) according to the manufacturer’s protocol (Invitrogen, Carlsbad, CA, USA) using Applied Biosystems 7900HT Real-Time PCR System. Cycle threshold (Ct) values were normalized by using glyceraldehyde-3-phosphate dehydrogenase (*Gapdh*) (TaqMan chemistry, Applied Biosystems) as a standard. Relative quantification was performed using the Livak method (28).

### Statistical analysis

All statistical analyses were performed in Statistica (data analysis software system), version 13.5 (TIBCO Software Inc.). The Shapiro-Wilk and Levene’s tests were used to verify the normal distribution and homogeneity of variances of the data, respectively. Morphometric and metabolic variables of the two groups were compared by unpaired Student *t*-test where *p*-value <0.05 was considered significant.

## Results

### Morphometric and metabolic profiles

**FIGURE 1:**
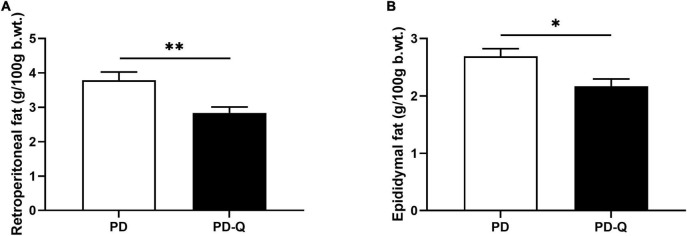
Effect of quercetin supplementation on retroperitoneal **(A)** and epididymal fat **(B)**. Values are mean ± SEM, *n* = 6/group, * *p* < 0.05, ** *p* < 0.01. PD, control rats; PD-Q, rats supplemented with quercetin; b.wt., body weight.

The effects of quercetin on morphometric and metabolic parameters in PD and PD-Q rats are shown in Table 1. No differences in rats’ food intake and total body weight during the experimental period were observed between the two tested groups. The relative weights of the liver and kidneys did not differ between PD and PD-Q rats. However, we detected increased heart and adrenal glands weights in rats after quercetin treatment (Table 1). In addition, PD-Q rats showed significantly lower relative weights of retroperitoneal and epididymal fat (Figures 1A,B), while no change in the weight of brown fat mass was detected between PD and PD-Q rats.

**TABLE 1:** Effect of quercetin supplementation on morphometric and metabolic variables in male PD rats.

Variables	PD	PD-Q	PD x PD-Q
**Morphometric variables**			
Initial body weight (g)	504 ± 13	489 ± 7	0.2
Final body weight (g)	532 ± 12	514 ± 8	0.1
Heart (g/100 g b.wt.)	0.25 ± 0.004	0.28 ± 0.01	0.03
Liver (g/100 g b.wt.)	2.99 ± 0.07	3.15 ± 0.06	0.2
Kidneys (mg/100 g b.wt.)	529 ± 13	542 ± 19	0.6
Adrenals (mg/100 g b.wt.)	10 ± 0.2	12 ± 0.5	0.01
Brown fat (mg/100 g b.wt.)	182 ± 10	193 ± 6	0.4
**Metabolic variables**			
Adiponectin (μg/ml)	2.63 ± 0.17	2.28 ± 0.15	0.3
Non-esterified fatty acids (mmol/L)	0.60 ± 0.04	0.54 ± 0.04	0.3

Variables are mean ± SEM, *n* = 6/group. PD, control rats; PD-Q, rats supplemented with quercetin; b.wt., body weight.

**FIGURE 2:**
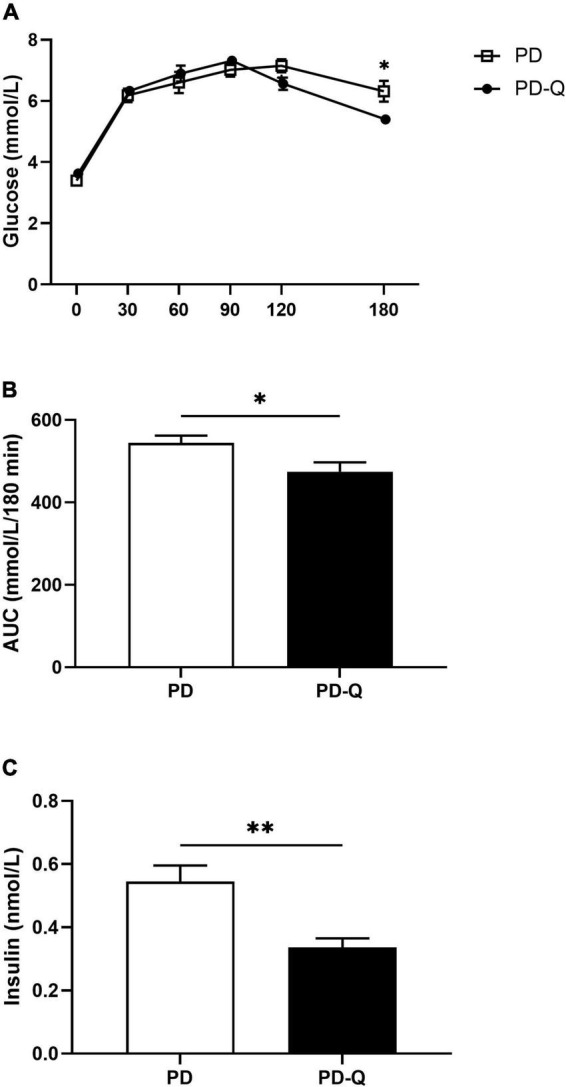
Effect of quercetin supplementation on blood glucose levels during oral glucose tolerance test **(A)**, area under the curve [AUC, **(B)**] and insulin levels **(C)**. Values are mean ± SEM, *n* = 6/group, * *p* < 0.05, ** *p* < 0.01, PD, control rats; PD-Q, rats supplemented with quercetin.

The levels of fasting blood glucose did not differ between PD and PD-Q rats. However, during the oral glucose tolerance test, PD-Q rats showed lower blood glucose level at the 180th min (Figure 2A). Furthermore, a smaller area under the glycemic curve and decreased fasting insulin concentration were detected in the PD-Q rats in comparison to the control group (Figures 2B,C).

**FIGURE 3:**
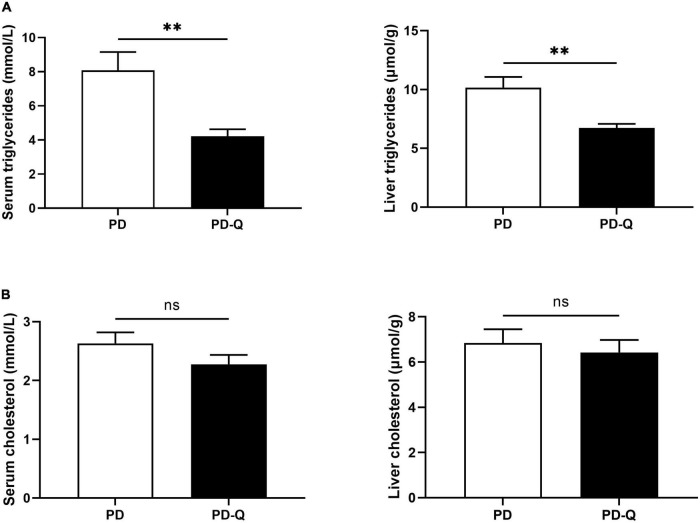
Effect of quercetin supplementation on the triglycerides **(A)** and cholesterol **(B)** levels in the serum and the liver tissue. Values are mean ± SEM, *n* = 6/group, ** *p* < 0.01, ns, not significant. PD, control rats; PD-Q, rats supplemented with quercetin.

The levels of adiponectin and free fatty acids did not differ between PD and PD-Q rats. Although no changes were detected in the serum and the liver cholesterol (Figure 3B), quercetin treatment significantly decreased the level of total triglycerides in the serum as well as in the liver tissue in PD-Q rats (Figure 3A).

### Transcriptomic analysis

**FIGURE 4:**
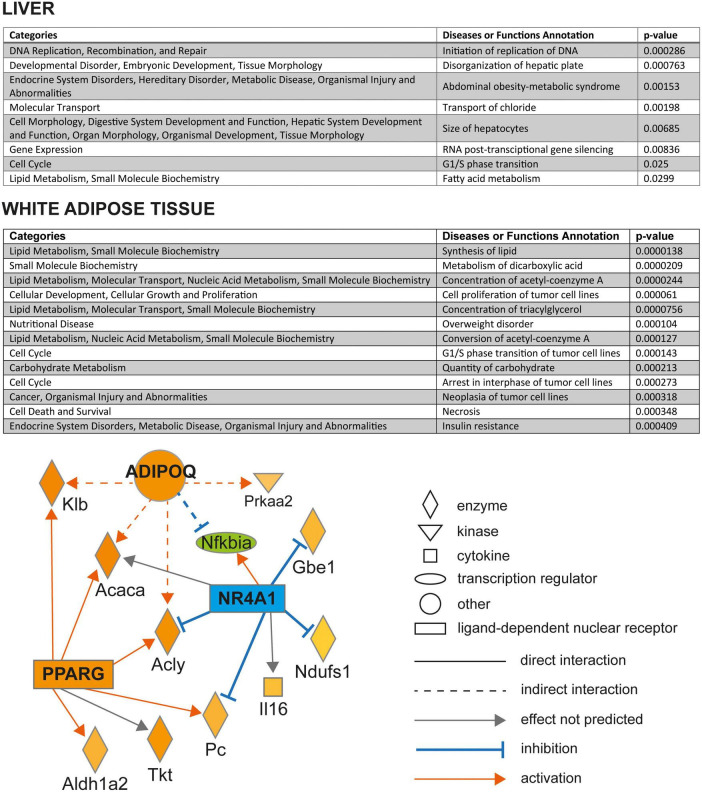
Overrepresented diseases and biological functions based on the differentially expressed genes in retroperitoneal adipose tissue of quercetin-treated vs. control PD rats in liver (**top panel**) and retroperitoneal adipose tissue (**middle panel**). The *p*-values (Fisher’s exact test) reflect the overrepresentation of differentially expressed genes in the particular disease and biological functions sets. **Bottom panel**: Mechanistic network of predicted upstream regulators (ADIPOQ, NR4A1, and PPARG) based on the dataset of differentially expressed genes in retroperitoneal adipose tissue of control and quercetin-treated PD rats. The quercetin effect on the expression of genes significantly differentially expressed is shown in shades of green (downregulation, blue for the predicted state of upstream regulators) or red (upregulation). All above analyses were performed using Ingenuity Pathways Analysis.

The list of all significantly differentially expressed transcripts in between PD and PD-Q rats in liver and retroperitoneal fat tissue is shown in Supplementary Tables 1A,B, respectively. There was no overlapping transcript between the sets of differentially expressed genes between adipose tissue and liver. In the adipose tissue, out of 32 transcripts showing significantly higher expression in PD-Q compared to PD, the most upregulated one was glycoprotein M6A (*Gpm6a*). On the other hand, only 16 transcripts were downregulated by quercetin, including microRNA-292, Stonin 1 (*Ston1*), or regulated endocrine specific protein 18 (*Resp18*). The canonical pathway analysis showed overrepresentation in four pathways, three of them inhibited - Ferroptosis signaling pathway (*p* = 0.011), LPS/IL-1 Mediated Inhibition of RXR Function (*p* = 0.016), and mitochondrial dysfunction (*p* = 0.024); the only activated canonical pathway was Thyroid hormone (TR)/RXR pathway activation (*p* = 0.037). The Diseases and Functions analysis pointed to the cellular processes in the adipose tissue most impacted by quercetin administration, mostly pertaining to the aspects of lipid metabolism, cell cycle, and the conditions relevant to metabolic syndrome (overweight disorder, insulin resistance) as shown in Figure 4. The upstream regulator analysis revealed six potentially activated or inhibited nodes (Supplementary Table 2A) affecting multiple of the differentially expressed genes in the adipose tissue. In particular, activated peroxisome proliferator-activated receptor gamma (*Pparg*), adiponectin (*Adipoq*), and inhibited state of nuclear receptor subfamily 4 group A member 1 (*Nr4a1*) formed a network consistent with the observed gene expression changes (Figure 4).

The comparison of hepatic transcriptomes of PD and PD-Q rats revealed only 20 transcripts to be significantly differentially expressed between the two groups after adjusting for multiple comparisons. Among the nine downregulated transcripts, the expression of zinc finger protein 354A (*Zfp354a*) and tsukushi, small leucine rich proteoglycan (*Tsku*) were most reduced by quercetin, while the genes most induced in livers of PD-Q *vs.* PD were ras homolog family member T1 (*Rhot1*) and amidohydrolase domain containing 1 (*Amdhd1*). The canonical pathway analysis did not reveal any overrepresented pathways in the liver based on the differentially expressed genes, most likely also due to their relatively low number. The Diseases and Functions analysis showed that quercetin affected the processes related to hepatocyte morphology, metabolic syndrome, cell cycle, RNA post-transcriptional gene silencing, and fatty acid metabolism (Figure 4). The upstream regulator analysis predicted inhibition of a proto-oncogene *Ets1* and of microRNA *Mir378* (Supplementary Table 2B). The expression of a selected subset of genes was validated by qPCR; in all cases, the differences in expression were corroborated (Supplementary Figure 1).

## Discussion

Metabolic syndrome (MetS) is a combination of cardiometabolic abnormalities, such as central obesity, hypertension, glucose intolerance and dyslipidemia, correlated with an increased risk for type 2 diabetes, cardiovascular disease, and all-cause mortality (2, 3). Quercetin, found naturally in numerous fruits and vegetables, is one of the most abundant flavonoids in the human diet (12). Extensive evidence has demonstrated its favorable impact on human health, including various features of MetS, such as obesity, glucose intolerance and dyslipidemia (17). In this study, we investigated the effects of quercetin on morphometric and metabolic parameters, as well as transcriptomic profiles in a highly inbred genetic rat model of MetS.

Even though the exact etiopathogenetic factors leading to the development of MetS remain to be elucidated, some investigators believe that an excess of abdominal (visceral) fat, being the most prevalent aspect of the syndrome, plays a major role in the process (3, 29). Quercetin has been previously shown to exert an anti-obesity effect due to its anti-inflammatory and antioxidant properties, thus decreasing abdominal fat mass and abdominal circumference (30–32). Likewise, in our study rats supplemented with quercetin showed lower weights of retroperitoneal and epididymal fat mass, both of which are considered a visceral fat. However, no changes in total body weight were detected between the two tested groups. Moreover, we detected an increased weights of the heart and adrenal tissues in quercetin treated rats compared to controls.

Individuals with increased visceral fat deposition also typically evince insulin resistance and hyperinsulinemia, resulting in impaired glucose tolerance (33, 34). In this study, we detected a significant decrease in the fasting insulin levels along with a lower postprandial blood glucose level at 180th min of the oral glucose tolerance test and smaller area under the glycemic curve in rats treated with quercetin. All these findings were expected based on extensive evidence demonstrating strong antidiabetic effect of quercetin in both animals and humans (14, 35, 36). In addition, some studies also described its possible beneficial effect in a prevention and treatment of long-term complications of diabetes, such as retinopathy, nephropathy, neuropathy, or atherosclerosis (36–39). Antidiabetic mechanisms of action of quercetin are pleiotropic and involve reducing insulin resistance, promoting insulin secretion, as well as inhibiting glucose absorption in the small intestine and/or improving glucose utilization in peripheral tissues (14, 40). Furthermore, quercetin also protects pancreatic β-cells against damage, preserves their mass and function, and stimulates regeneration of β-cells (41, 42).

Atherogenic dyslipidemia, namely high triglycerides and low HDL-cholesterol levels, is an integral component of MetS and a major risk factor for developing cardiovascular diseases (43, 44). Although quercetin seems to have a favorable effect toward normalizing blood lipid levels, the results are not consistent. Many studies report very little or no improvement in lipid levels after quercetin administration (45, 46). In this study, we detected no significant changes in total cholesterol and free fatty acid levels. However, the level of triglycerides (TG) greatly decreased in the serum as well as in the liver of rats treated with quercetin. Some other studies also support these findings confirming the ability of quercetin to decrease TG levels, while total cholesterol levels remained unchanged or even increased at the end of the experimental period (46, 47). Several mechanisms leading to a decrease in the TG levels were described, such as increased TG-derived uptake of fatty acids by white adipose tissue as a consequence of browning and/or modulating gut microbiota (48, 49). Further studies are needed to fully elucidate the effect of quercetin on blood lipids levels and its clinical relevance.

One of the most quercetin-downregulated genes in liver was tsukushi (*Tsku*). It was identified as an inducible hepatokine, its deficiency protected mice from high-fat-diet-induced obesity and metabolic disorders and was connected with reduced adiposity (50), similarly to the effects observed in quercetin-fed rats in this study. While the exact function of the most downregulated hepatic gene, *Zfp354a*, is not clear, it was found to have cis-expression quantitative trait locus (eQTL) and, at the same time, a significant correlation between its expression in liver or adipose tissue with hepatic TG levels in mouse model of hepatic steatosis (51). Also, its hepatic expression increases after ethanol bolus (52). Quercetin-fed rats had substantially increased expression of a mitochondrial Rho-GTPase, *Rhot1*, crucial for mitochondrial trafficking and peroxisomal fission (53). The nodes identified in upstream regulator analysis of the retroperitoneal adipose tissue transcriptome revealed the potentially beneficial shifts in gene expression.

There is ample evidence for the involvement of microRNAs in both pathogenesis of metabolic syndrome and the favorable action of quercetin and other polyphenols (54). Here, the most downregulated transcript by quercetin in the adipose tissue is *Mir292*, and in the liver, *Mir378* was identified as an important inhibited upstream regulator. *Mir292* belongs to the microRNA cluster, which is specific to the pluripotent stem cells and promotes rapid G1/S phase transition within the cell cycle (55). The observed lower *Mir292* expression may thus reflect the deceleration of the cell cycle in adipose tissue stem cells in the situation of quercetin-induced fat loss. *Mir378* is encoded within a master metabolic regulator, the peroxisome proliferator-activated receptor γ coactivator 1β (PGC-1β), and mice lacking *Mir378* were resistant to high-fat diet-induced obesity and exhibited enhanced mitochondrial fatty acid metabolism and elevated oxidative capacity of insulin-target tissues (56). Together with the predicted upregulation of *Adipoq*, *Pparg* and *Ppargc1*a and downregulation of *Nos2* nodes, the overall transcriptome shift corresponds to a gene expression profile repeatedly associated with amelioration of insulin resistance (57–60). This could represent the potential underlying mechanism of the observed insulin-sensitizing action in quercetin-treated rats.

The limitations of our study include the use of only adult male rats of a single inbred strain as sex-specific genetic architecture of MetS and its components (61). The experimental design aimed to address the subtle effects of short-term quercetin administration, therefore, we opted to perform the experiment while maximizing the homogeneity of the experimental and control groups. In addition, a single dose and regimen of quercetin administered to the model animals did not allow us to assess dosage-dependent effects.

## Conclusion

Individual features of MetS, such as adiposity, glucose intolerance and blood triglycerides levels in rats can be ameliorated by quercetin. Here, we present crucial transcriptomic nodes and networks, through which the quercetin may effectuate its metabolic actions on liver and adipose tissue. As the prevalence of MetS is still increasing, dietary supplementation with either purified quercetin or food rich in quercetin could potentially be an effective intervention strategy.

## Data availability statement

The datasets presented in this study can be found in the ArrayExpress repository under accession number: E-MTAB-11061/Supplementary material.

## Ethics statement

The animal study was reviewed and approved by Ethics Committees of the First Faculty of Medicine of the Charles University.

## Author contributions

AK and OŠ contributed to conception and design of the study and involved in data curation, statistical analysis, and funding acquisition. HM, IM, and MH carried out the metabolic profiling. AK and BC performed transcriptomic analysis and real-time PCR. AK drafted the initial manuscript. OŠ, BC, HM, IM, and MH participated in writing—review and editing the manuscript. All authors contributed to manuscript revision, read, and approved the submitted version.

## Footnotes

1
https://www.ebi.ac.uk/arrayexpress/
