# Supplementary material for: Quercetin supplementation alters adipose tissue and hepatic transcriptomes and ameliorates adiposity, dyslipidemia, and glucose intolerance in adult male rats
Source: Front Nutr. 2022 Sep 29;9:952065. doi: 10.3389/fnut.2022.952065 (PMC9558266; doi:10.3389/fnut.2022.952065)
Supplement: Supplementary file 1 [file Table_1.DOCX]

**Table S2A** Effect of quercetin supplementation on upstream regulators in retroperitoneal adipose tissue.

| Upstream Regulator | Predicted Activation State | Activation z-score | p-value |
| --- | --- | --- | --- |
| ADIPOQ | Activated | 2.4 | 0.000364 |
| PPARG | Activated | 2.4 | 0.000751 |
| PPARGC1A | Activated | 2.2 | 0.00699 |
| NOS2 | Inhibited | -2 | 0.00583 |
| NR4A1 | Inhibited | -2.2 | 0.000113 |
| TP53 | Inhibited | -3.9 | 0.000949 |

**Table S2B** Effect of quercetin supplementation on upstream regulators in liver.

| Upstream Regulator | Predicted Activation State | Activation z-score | p-value |
| --- | --- | --- | --- |
| Mir-378 | Inhibited | -1.3 | 0.0083 |
| ETS1 | Inhibited | -1.4 | 0.0037 |

**Legend:** the upstream regulators were identified using Ingenuity Upstream Regulator Analysis. The overlap p-value, calculated using Fisher’s exact test, reflects whether there is a statistically significant overlap between the dataset genes and the genes that are regulated by a transcriptional regulator. The activation z-score is computed to infer the activation states of predicted transcriptional regulators. ADIPOQ: adiponectin, PPARG: peroxisome proliferator activated receptor gamma, PPARGC1A: PPARG coactivator 1 alpha, nitric oxide synthase 2, NR4A1: nuclear receptor subfamily 4 group A member 1, TP53 tumor protein p53, Mir-378: microRNA-378, ETS1: ETS proto-oncogene 1, transcription factor.
