# Supplementary material for: Quercetin supplementation alters adipose tissue and hepatic transcriptomes and ameliorates adiposity, dyslipidemia, and glucose intolerance in adult male rats
Source: Front Nutr. 2022 Sep 29;9:952065. doi: 10.3389/fnut.2022.952065 (PMC9558266; doi:10.3389/fnut.2022.952065)
Supplement: Supplementary file 2 [file Data_Sheet_1.PDF]

Supplementary Table S1.

The summary of significantly differentially expressed transcripts between PD-Q (quercetin-treated) and PD (control) adult male rats

A. The summary of all significantly differentially expressed transcripts in liver

Affymetrix Transcript ID	Gene Symbol	Gene name	p-value (PD-Q vs. PD)	Fold-Change (PD-Q vs. PD)
17645579	*Zfp354a*	zinc finger protein 354A	5.58E-05	-2.47
17635099	*Tsku*	tsukushi, small leucine rich proteoglycan	1.59E-04	-1.82
17839371	*Tob2*	transducer of ERBB2, 2	5.00E-05	-1.35
17797880	*Gabrr1*	gamma-aminobutyric acid type A receptor subunit rho1	2.17E-04	-1.24
17635713	*Olr170*	olfactory receptor 170	3.47E-04	-1.23
17719143	*Wrnip1*	WRN helicase interacting protein 1	3.28E-04	-1.22
17808107	*Brinp1*	BMP/retinoic acid inducible neural specific 1	1.84E-04	-1.22
17639781	*Pola2*	DNA polymerase alpha 2, accessory subunit	2.33E-05	-1.21
17614203	*Zfp428*	zinc finger protein 428	1.59E-04	-1.20
17872007	*Clcn4*	chloride voltage-gated channel 4	3.59E-04	1.20
17688961	*RGD1561381*	similar to microsomal glutathione S-transferase 3	1.45E-04	1.20
17730899	*Spata33*	spermatogenesis associated 33	3.06E-04	1.27
17614842	*Dyrk1b*	dual specificity tyrosine phosphorylation regulated kinase 1B	3.19E-04	1.28
17802830	*Xkr8*	XK related 8	1.02E-04	1.29
17779108	*Ada*	adenosine deaminase	2.16E-05	1.29
17622570	*Dhcr7*	7-dehydrocholesterol reductase	3.59E-04	1.30
17723779	*St8sia3*	ST8 alpha-N-acetyl-neuraminide alpha-2,8-sialyltransferase 3	2.13E-04	1.33
17743211	*Gbp3*	guanylate binding protein 3	5.35E-05	1.64
17835248	*Amdhd1*	amidohydrolase domain containing 1	1.57E-04	1.85
17649347	*Rhot1*	ras homolog family member T1	3.69E-04	2.21

B. The summary of all significantly differentially expressed transcripts in retroperitoneal adipose tissue

Affymetrix Transcript ID	Gene Symbol	Gene name	p-value (PD-Q vs. PD)	Fold-Change (PD-Q vs. PD)
17711575	*Gpm6a*	glycoprotein M6A	1.83E-04	4.38
17849839	*Muc16*	mucin 16, cell surface associated	6.89E-07	4.08
17829240	*Pkhd1l1*	PKHD1 like 1	4.70E-04	3.55
17676916	*Upk3b*	uroplakin 3B	2.71E-05	2.95
17654839	*Msln*	mesothelin	2.18E-04	2.89
17615434	*Sbsn*	suprabasin	1.70E-04	2.12
17649706	*Acaca*	acetyl-CoA carboxylase alpha	1.83E-04	2.10
17694472	*Klb*	klotho beta	3.63E-04	1.96
17664369	*Cxadr*	CXADR Ig-like cell adhesion molecule	3.43E-05	1.72
17661380	*Acly*	ATP citrate lyase	8.20E-05	1.69
17852732	*Chrnb4*	cholinergic receptor nicotinic beta 4 subunit	7.65E-05	1.58
17705975	*Tkt*	transketolase	3.64E-04	1.49
17870839	*Maob*	monoamine oxidase B	1.00E-04	1.46
17845663	*Aldh1a2*	aldehyde dehydrogenase 1 family member A2	7.01E-05	1.43
17742837	*Slc9b2*	solute carrier family 9 member B2	2.96E-05	1.43
17858614	*Npas2*	neuronal PAS domain protein 2	3.12E-04	1.40
17664254	*Gbe1*	1,4-alpha-glucan branching enzyme 1	3.56E-04	1.38
17710569	*Pc*	pyruvate carboxylase	6.82E-06	1.36
17619189	*Olr168*	olfactory receptor 168	2.12E-04	1.36
17634604	*Il16*	interleukin 16	1.50E-05	1.34
17644592	*Tpsb2*	tryptase beta 2	1.87E-04	1.32
17624288	*Glyatl1*	glycine-N-acyltransferase like 1	9.31E-05	1.29
17692287	*Gpr75*	G protein-coupled receptor 75	3.88E-04	1.27
17676630	*Vom2r64*	vomeronasal 2 receptor, 64	2.47E-04	1.25
17865002	*Ndufs1*	NADH:ubiquinone oxidoreductase core subunit S1	1.22E-04	1.24
17808996	*Prkaa2*	protein kinase AMP-activated catalytic subunit alpha 2	3.53E-04	1.23
17737018	*Zfp458*	zinc finger protein 458	1.32E-04	1.22
17658462	*Aspa*	aspartoacylase	3.03E-04	1.22
17845185	*Clpx*	caseinolytic mitochondrial matrix peptidase chaperone subunit X	3.87E-04	1.21
17725547	*Hspa9*	heat shock protein family A (Hsp70) member 9	1.45E-04	1.20
17867686	*LOC365085*	similar to nidogen 2	3.88E-04	1.20
17731159	*Disc1*	DISC1 scaffold protein	4.44E-04	1.20
17826729	*Gm9978*	predicted gene 9978	1.73E-04	-1.21
17732209	*Gpt2*	glutamic--pyruvic transaminase 2	1.84E-04	-1.21
17765471	*Serf2*	small EDRK-rich factor 2	1.87E-04	-1.23
17774363	*Olr714*	olfactory receptor 714	1.69E-04	-1.23
17639871	*Snx15*	sorting nexin 15	2.99E-04	-1.24
17756442	*Ddah2*	dimethylarginine dimethylaminohydrolase 2	3.50E-04	-1.24
17796618	*Chmp4bl1*	chromatin modifying protein 4B-like 1	1.22E-04	-1.26
17821997	*Nfkbia*	NFKB inhibitor alpha	7.58E-05	-1.27
17646778	*Hs3st3a1*	heparan sulfate-glucosamine 3-sulfotransferase 3A1	4.01E-04	-1.28
17870134	*Irx1*	iroquois homeobox 1	5.05E-06	-1.33
17880467	*Tuba1b*	tubulin alpha 1b	1.81E-04	-1.39
17865716	*Resp18*	regulated endocrine specific protein 18	4.39E-04	-1.40
17813989	*Ston1*	stonin 1	1.24E-04	-1.40
17739833	*Paqr6*	progestin and adipoQ receptor family member 6	2.45E-04	-1.42
17766478	*Prnp*	prion protein	2.51E-04	-1.45
17629284	*Mir292*	microRNA 292	9.47E-05	-1.46
