# Supplementary material for: Quercetin supplementation alters adipose tissue and hepatic transcriptomes and ameliorates adiposity, dyslipidemia, and glucose intolerance in adult male rats
Source: Front Nutr. 2022 Sep 29;9:952065. doi: 10.3389/fnut.2022.952065 (PMC9558266; doi:10.3389/fnut.2022.952065)
Supplement: Supplementary file 3 [file Data_Sheet_2.PDF]

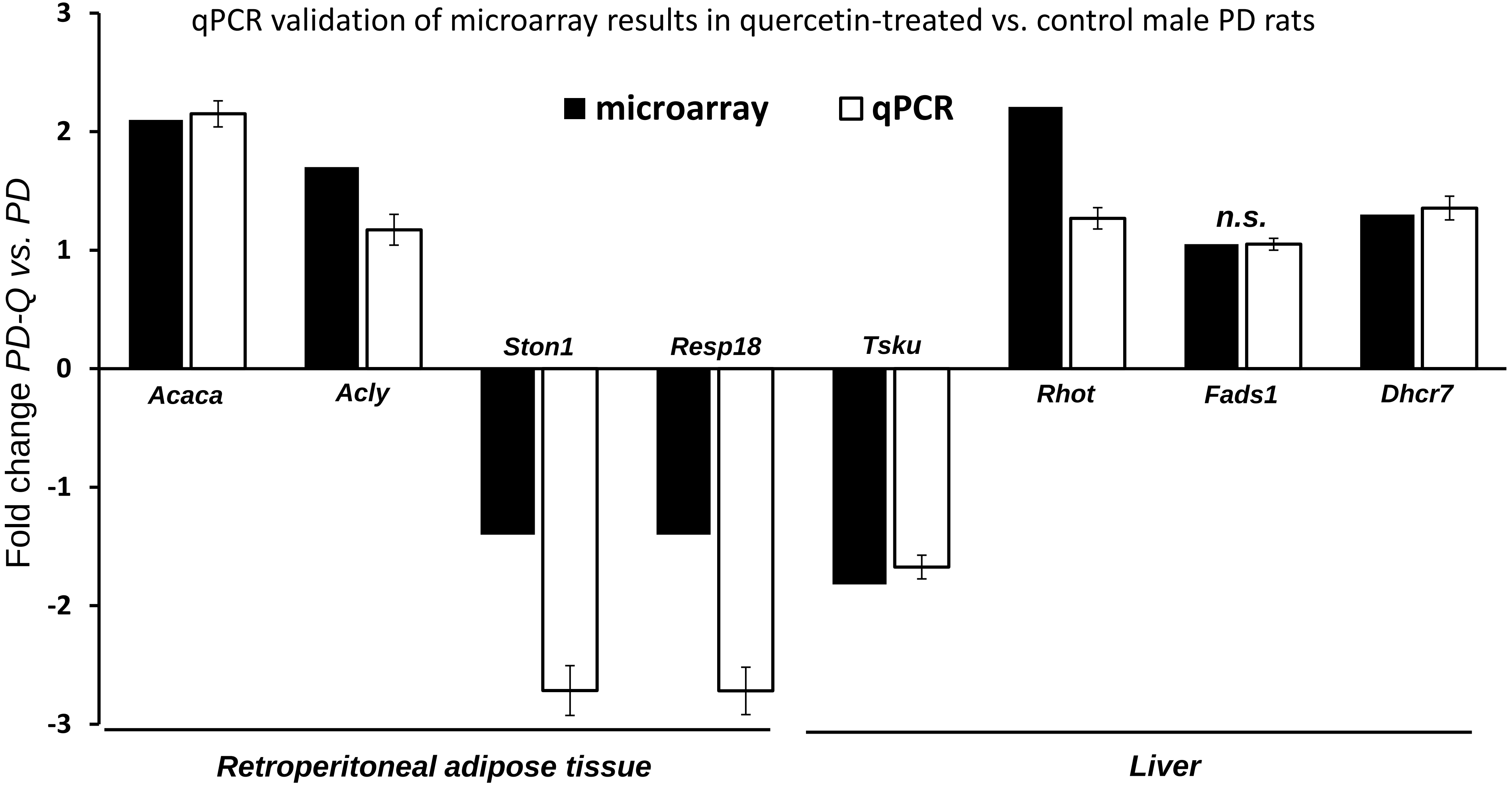

Figure S1: Validation of microarray results using qPCR. Fold changes are indicated for quercetin effect in adult PD male rats, i.e. comparison of the gene expression in quercetin-treated (PD-Q) vs. control (PD) groups (microarray (Rat Gene 2.1 ST Array): black bars; qPCR: white bars). *Acaca*: acetyl-CoA carboxylase alpha; *Acly*: ATP citrate lyase, *Ston1*: Stonin 1; *Resp18*: regulated endocrine specific protein 18; *Tsku*: tsukushi, small leucine rich proteoglycan; *Rhot1*: ras homolog family member T1; , *Fads1*: fatty acid desaturase 1; *Dhcr7*: 7-dehydrocholesterol reductase.
